# Comprehensive study on the efficiency of vertical bifacial photovoltaic systems: a UK case study

**DOI:** 10.1038/s41598-024-68018-1

**Published:** 2024-08-08

**Authors:** Ghadeer Badran, Mahmoud Dhimish

**Affiliations:** School of Physics, Engineering and Technology, University of York, York, YO10 5DD UK

**Keywords:** Renewable energy, Solar energy, Photovoltaics

## Abstract

This paper presents the first comprehensive study of a groundbreaking Vertically Mounted Bifacial Photovoltaic (VBPV) system, marking a significant innovation in solar energy technology. The VBPV system, characterized by its vertical orientation and the use of high-efficiency Heterojunction cells, introduces a novel concept diverging from traditional solar panel installations. Our empirical research, conducted over a full year at the University of York, UK, offers an inaugural assessment of this pioneering technology. The study reveals that the VBPV system significantly outperforms both a vertically mounted monofacial PV (VMPV) system and a conventional tilted monofacial PV (TMPV) system in energy output. Key findings include a daily power output increase of 7.12% and 10.12% over the VMPV system and an impressive 26.91% and 22.88% enhancement over the TMPV system during early morning and late afternoon hours, respectively. Seasonal analysis shows average power gains of 11.42% in spring, 8.13% in summer, 10.94% in autumn, and 12.45% in winter compared to the VMPV system. Against the TMPV system, these gains are even more substantial, peaking at 24.52% in winter. These results underscore the VBPV system's exceptional efficiency in harnessing solar energy across varied environmental conditions, establishing it as a promising and sustainable solution in solar energy technology.

## Introduction

Solar photovoltaic (PV) technology has become a cornerstone of the renewable energy revolution, offering a clean, sustainable solution to the world's growing energy demands^1^. At its core, solar PV harnesses the sun's energy, converting it directly into electricity through semiconducting materials. This technology has traditionally been dominated by monofacial PV modules^2^, which collect sunlight from a single surface facing the sun. However, as the need for more efficient and cost-effective energy solutions intensifies, the evolution of solar PV has given rise to the bifacial module^3,4^—a novel approach to solar energy capture that promises to redefine the efficiency standards of solar energy systems.

**Figure. 1.**
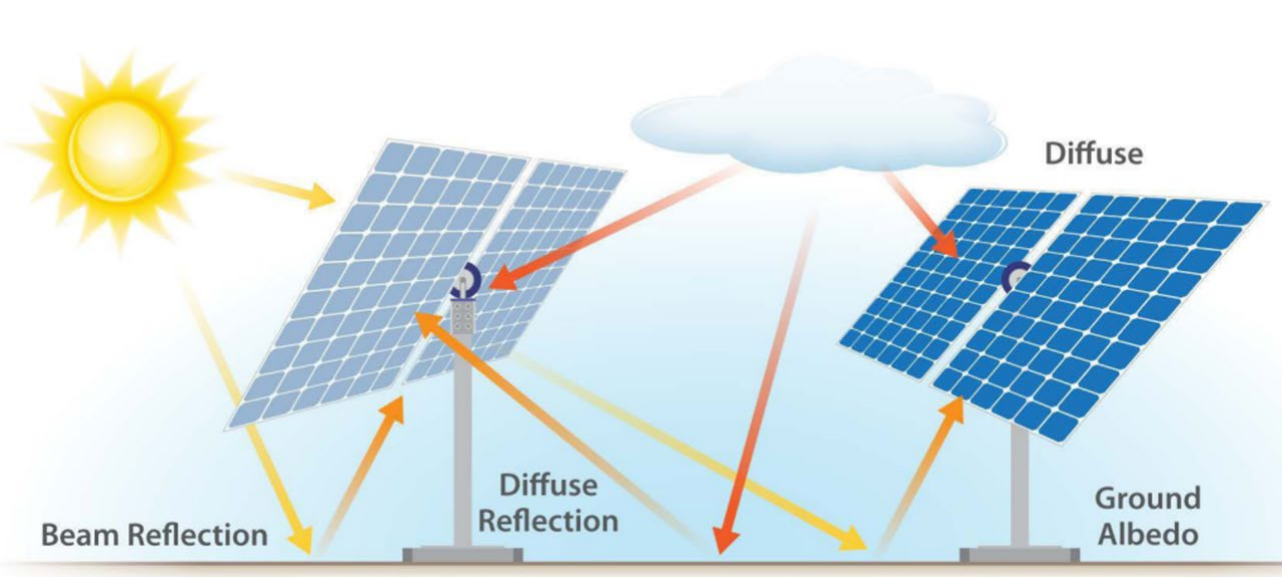
Illustration of bifacial PV system operation. The arrows indicate the different pathways of sunlight: yellow arrows represent direct sunlight hitting the front surface and the ground, orange arrows indicate the sunlight reflected from the ground hitting the rear surface, and red arrows depict the diffuse sunlight captured by both the front and rear surfaces ^11^.

Bifacial PV modules, as shown in Fig. 1, are designed to capture sunlight on both their front and rear surfaces, utilizing direct sunlight and the light that reaches the rear surface through ground reflection and diffuse albedo^5,6^. Despite relying on silicon cells with the same spectral response as monofacial PV modules, the dual-sided design of bifacial modules allows them to significantly enhance energy yield by absorbing reflected and diffused light from surrounding surfaces^7^. This design is particularly beneficial in environments with high ground reflectivity or engineered ground covers to increase reflectivity^8^.

The evolution of bifacial PV modules represents more than just an incremental improvement in solar technology; it signifies a paradigm shift in how solar energy is harvested. Unlike traditional monofacial systems^9^ that are limited by their unidirectional light capture, bifacial systems exploit the full spectrum of solar irradiance. This is achieved through a combination of advanced cell technology and innovative panel designs, which optimize light absorption from multiple angles^10^. The result is a marked increase in energy production per unit area, a critical factor in maximizing the efficiency of solar installations.

Moreover, the integration of bifacial PV technology aligns seamlessly with the global push towards sustainable development. By enhancing the power output of solar installations without the need for additional land, bifacial PV systems contribute to a more efficient use of resources. This efficiency is not confined to optimal conditions; bifacial modules demonstrate resilience in a variety of environmental settings^11,12^, including regions with lower solar irradiance and urban landscapes^13^ where space and light conditions are constrained.

The significance of bifacial PV modules extends beyond their operational advantages. Their deployment has profound implications for energy policy, economic planning, and environmental strategy. By offering a more versatile and powerful solution for solar energy generation, bifacial PV systems can accelerate the transition to renewable energy sources, reduce dependency on fossil fuels, and mitigate the impacts of climate change.

In the realm of bifacial PV technology, various configurations have been explored to maximize the efficiency and adaptability of solar energy systems. These include vertical, tilted, and other innovative arrangements, each with its unique operational characteristics and applications. Vertical bifacial PV systems: These systems involve panels mounted in a vertical orientation. The key advantage of vertical bifacial PV is its ability to capture sunlight effectively throughout the day, from both sides of the panel^14^. This configuration is particularly beneficial in higher latitudes where the sun is lower in the sky^15^. Vertical systems are also less prone to accumulating dirt and debris, reducing maintenance requirements. Current research indicates that vertical bifacial systems can achieve significant energy gains in urban environments, where space is limited, and in regions with considerable diffuse light^16^.

Tilted bifacial PV Systems: Tilted systems are more traditional, where panels are installed at an angle to maximize exposure to direct sunlight. Bifacial panels in this configuration can capture reflected light from the ground or any reflective surface below. The optimal tilt angle is a subject of ongoing research, with studies^17–19^ suggesting that slight adjustments in the tilt can lead to substantial increases in energy capture, particularly in areas with high ground albedo. And finally, tracking bifacial PV systems: These are dynamic systems where panels can adjust their orientation to follow the sun’s path^20^. This tracking capability, combined with bifacial technology, maximizes solar energy capture throughout the day. Research^21,22^ shows that tracking bifacial systems offer the highest yield, especially in regions with high direct sunlight, making them a promising solution for large-scale solar farms.

Each of these configurations brings unique advantages and challenges, shaping the current research and development in the field of bifacial PV technology. Studies are continually underway to optimize the design, installation, and operational parameters of these systems. This includes investigating factors like the optimal distance between rows of panels^23^ to prevent shading, the effect of different surfaces^24^ and materials on light reflection, and the integration of smart technologies for performance monitoring and optimization. Furthermore, the performance of bifacial PV systems is significantly influenced by shading and the reflective properties of surrounding surfaces. Shading can reduce the overall efficiency by blocking sunlight from reaching both the front and rear surfaces of the panels. Detailed models of shading and illumination, such as those reported by^25^ and^26^, provide comprehensive insights into these effects. In^25^ the authors demonstrated that partial shading could lead to substantial reductions in energy output, especially in high-density installations. Further work by^26^ explored the impacts of various surface materials and albedo on bifacial PV performance, showing that engineered surfaces with higher reflectivity can enhance energy yield by increasing the diffuse light captured by the rear surface of the panels. These models underscore the importance of considering shading and surface properties in the design and deployment of bifacial PV systems to optimize their performance.

The evolution of bifacial PV modules represents more than just an incremental improvement in solar technology; it signifies a paradigm shift in how solar energy is harvested. Unlike traditional monofacial systems that are limited by their unidirectional light capture, bifacial systems exploit the full spectrum of solar irradiance. This is achieved through a combination of advanced cell technology and innovative panel designs, which optimize light absorption from multiple angles. While Heterojunction (HJT) cells are a prominent technology used in bifacial modules, other technologies such as n-type^27^, Passivated Emitter and Rear Cell (PERC)^28^, Passivated Emitter Rear Totally Diffused (PERT)^29^, Passivated Emitter Rear Locally Diffused (PERL)^30^, and Interdigitated Back Contact (IBC)^30^ solar cells are also suitable for bifacial applications, demonstrating widely successful results. These technologies collectively contribute to the marked increase in energy production per unit area^31^, a critical factor in maximizing the efficiency of solar installations.

This study introduces the first-ever exploration and publication on the vertically mounted bifacial photovoltaic (VBPV) system, a groundbreaking advancement in solar energy technology. This prototype's uniqueness stems from its vertical orientation and the use of high-efficiency Heterojunction (HJT) cells, a significant departure from traditional solar panel setups. Our research is pioneering in its empirical approach, offering the initial real-world evaluation of the VBPV system's performance across various environmental conditions over an entire year. This includes a comparative analysis with conventional monofacial systems, providing new insights into the practical efficiencies and benefits of bifacial technology. Additionally, the study navigates the complexities of modelling such an innovative system, addressing the challenges in accurately predicting performance and highlighting the need for advanced simulation techniques.

## Materials and methods

### New vertical PV bifacial concept design

**Figure 2:**
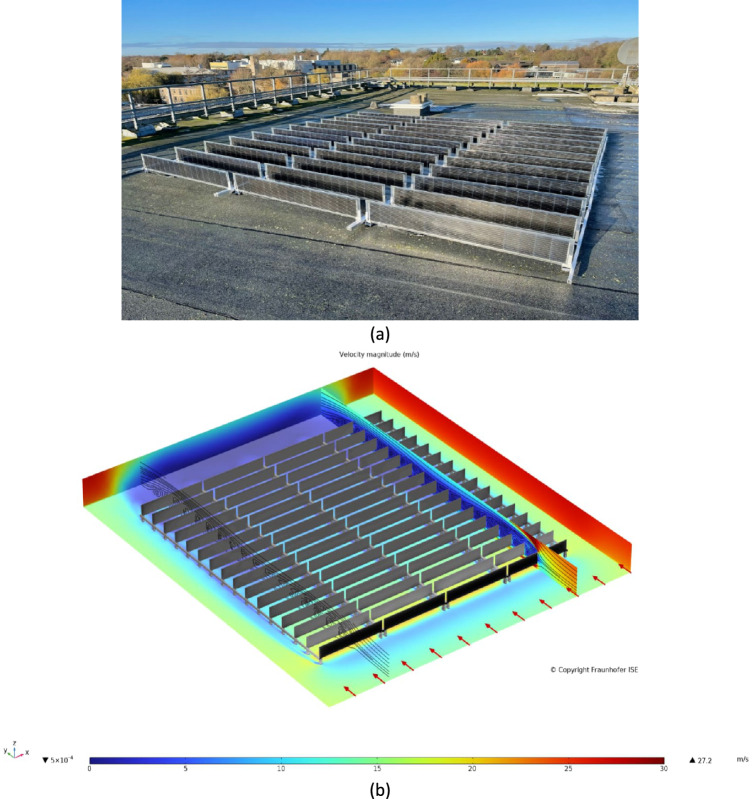
The new VBPV system examined in this work. (**a**) The system is located on the rooftop of the Physics Tower at the University of York, UK. The ground surface material is white gravel, chosen to enhance the albedo effect and increase the diffuse reflection captured by the rear side of the bifacial panels, (**b**) CFD simulation of the VBPV system under examination in this work, indicating the system has negligible lift forces at extreme wind speeds of 27.2 m/s.

This study presents a pioneering exploration and evaluation of the vertically mounted bifacial photovoltaic system, focusing on its unique design and operational characteristics. The VBPV system utilizes high-efficiency HJT cells and is mounted in a vertical orientation, which significantly differs from traditional solar panel setups^32,33^. The experimental setup involved the installation of the VBPV system on the rooftop of the Physics Tower at the University of York (Fig. 2a). The system comprises 36 series-connected PV units with a maximum output power of 1.5 kW under standard test conditions (STC) of 1000 W/m^2^ irradiance and 25 °C ambient temperature. The location of the system was selected to maximize exposure to sunlight while also taking advantage of the reflective properties of the surrounding environment. The ground surface material beneath and around the PV modules is white gravel, known for its high albedo. This choice of material enhances the diffuse reflection, thereby increasing the amount of light captured by the rear side of the bifacial panels and boosting the overall energy yield. This setup ensures that the system benefits from both direct and reflected sunlight, optimizing its performance across various environmental conditions.

The distance between each row of modules is 50 cm. This spacing was determined based on extensive simulations by Over Easy Solar AS, Norway, to optimize the balance between minimizing shading and maximizing ground reflection. This decision, while not arbitrary, aligns with findings from other research indicating that the optimal distance is a function of module height and should be carefully considered for each specific installation^34–36^. In addition to the nominal power output, the system's performance characteristics include a temperature coefficient of −0.29%/°C and a conversion efficiency of 22.5%, which are critical for understanding the operational efficiency and resilience of the VBPV system under varying environmental conditions.

The performance of the VBPV system was continuously monitored over a full annual cycle, from February 2023 to December 2023, and compared against a vertically mounted monocrystalline silicon monofacial PV (VMPV) system and a traditional tilted monofacial PV (TMPV) system. Data was recorded using a 3-kW inverter integrated with the university's grid, allowing for real-time tracking and analysis of energy production. This comprehensive empirical approach provides valuable insights into the practical efficiencies and benefits of bifacial technology, highlighting the superior performance of the VBPV system under varied environmental conditions.

The VBPV system was subjected to a Computational Fluid Dynamics (CFD) simulation to assess its aerodynamic stability. The simulation was conducted using ANSYS Fluent, employing a k-ε turbulence model to accurately capture the airflow dynamics around the panels. The boundary conditions included an inlet wind speed of up to 27 m/s, representing extreme weather conditions that the system might encounter. The panels were modeled with a surface roughness corresponding to the actual material properties, and the spacing between panels was set at 50 cm, as per the physical setup.

The CFD simulation results, shown in Fig. 2b, reveal that the VBPV system maintains minimal lift forces even at high wind speeds of up to 27 m/s. This indicates exceptional aerodynamic stability, which is crucial for ensuring the durability and safety of the installation in adverse weather conditions. In comparison, traditional tilted PV systems have been documented to experience higher lift forces under similar wind conditions due to their inclined surfaces which can act like airfoils.

### Data comparison and analysis

The innovative VBPV system under study is strategically positioned on the rooftop of the Physics Tower at the University of York, UK. It has been meticulously oriented towards the south to optimize solar gain. This system is seamlessly integrated with a 3-kW inverter, which facilitates the direct feed of generated electricity into the university's grid. The performance data of the system is meticulously monitored and recorded through the inverter's online platform, ensuring real-time tracking and analysis of energy production.

The installation of the VBPV system was completed in December 2022, with its official commissioning taking place in January 2023. As such, the performance data presented and analyzed in this work encompasses a comprehensive annual cycle, ranging from February 2023 to the end of December 2023. This dataset provides a robust foundation for assessing the system’s efficiency and energy output across various seasonal conditions.

**Figure 3:**
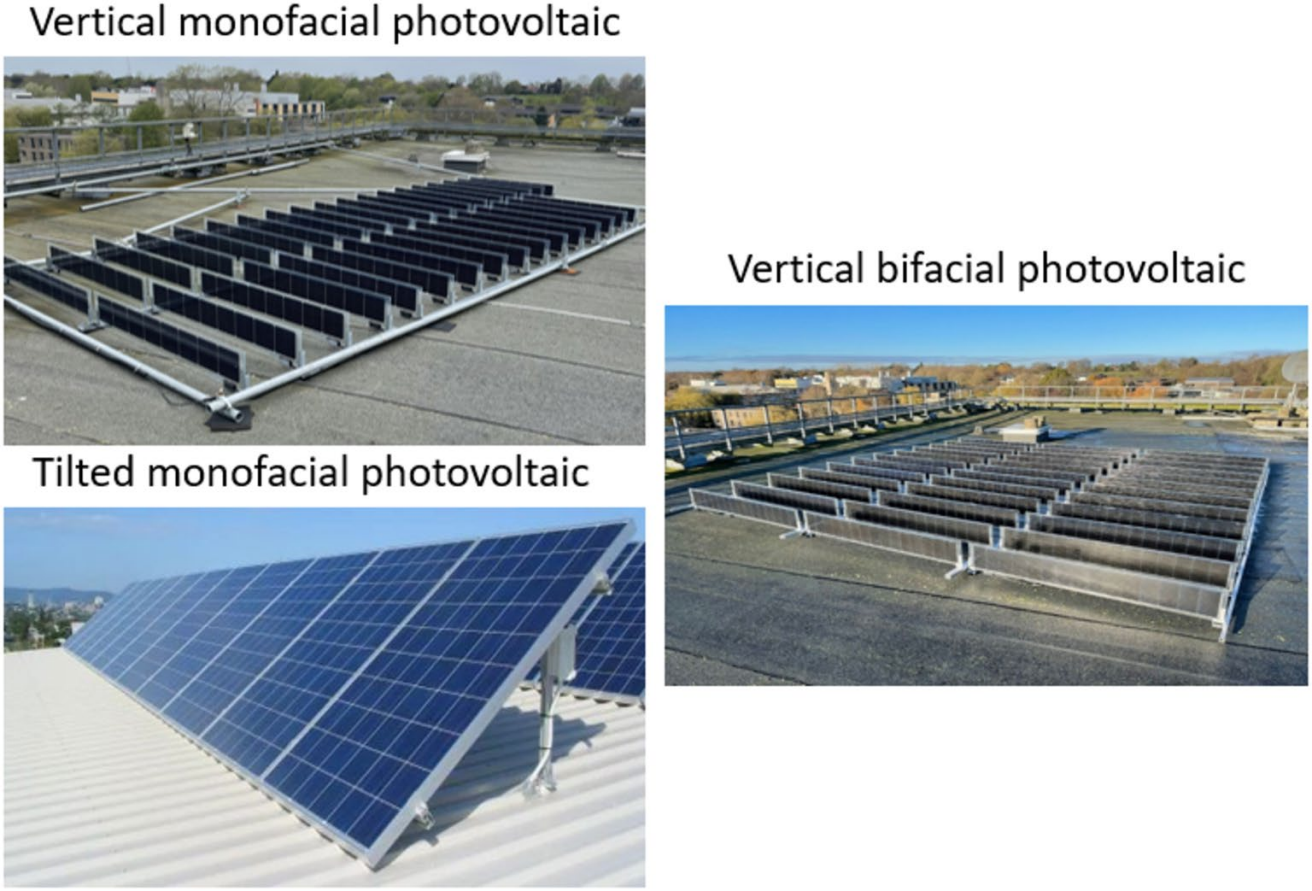
Comparison of Three Examined Photovoltaic (PV) System Configurations.

To establish a baseline for comparison and underscore the VBPV system's performance, we juxtaposed its data against that of a vertically mounted monocrystalline silicon monofacial PV (VMPV) system situated adjacent to it, with the same PV capacity of 1.5 kW. This parallel analysis illuminates the advantages of bifacial technology in a like-for-like vertical setup. Furthermore, to extend the comparative analysis, we scrutinized the VBPV system's output relative to that of a traditional tiled 1.5 kW polycrystalline silicon monofacial PV system (TMPV). The latter is installed at the customary 45-degree angle prevalent in UK solar installations, thus representing the conventional approach to solar energy generation in the region; all PV configurations examined in this work are presented in Fig. 3.

The power gain between two PV systems, such as the VBPV compared to VMPV or TMPV, is calculated using (1).

1 $$ Power\;Gain\; Percentage = \frac{{Power\; Output_{VBPV} - Power \;Output_{Reference \;System} }}{{Power\; Output_{Reference\; System} }} \times 100\% $$

where $Power\; Output_{VBPV}$ is the electrical power output of the VBPV, and $Power \;Output_{Reference\; System}$ is the electrical power output of the reference system, which can be either VMPV or TMPV.

## Results

### Vertical bifacial PV vs vertical monofacial PV

**Figure 4:**
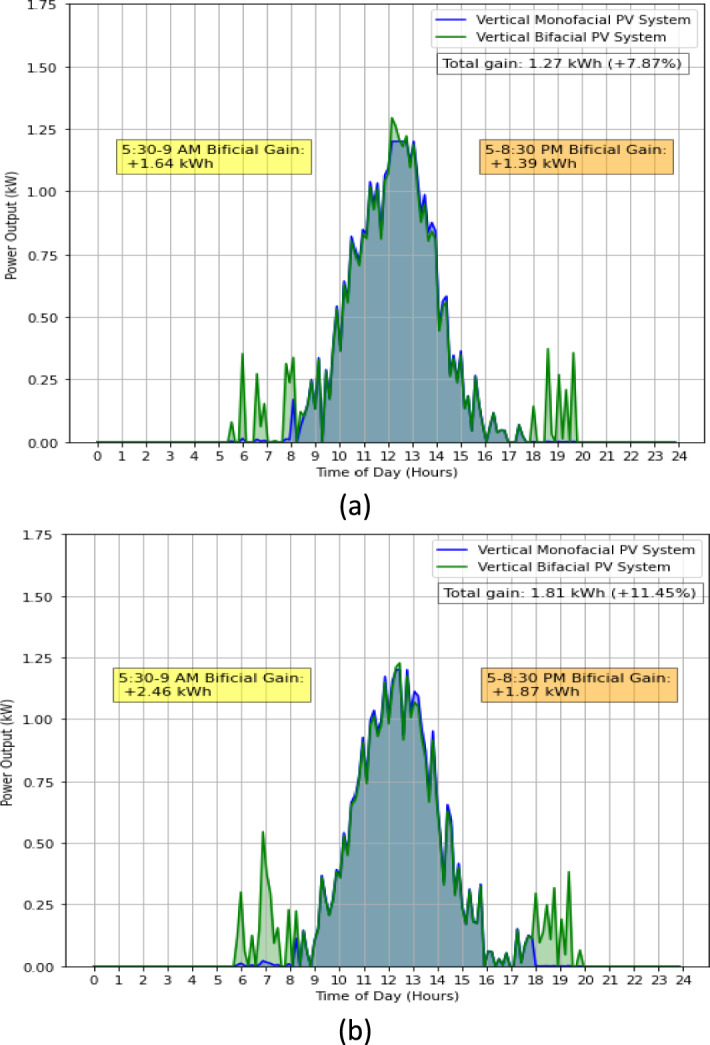
Comparative daily power output of VBPV versus VMPV Systems, highlighting bifacial gain in early morning and late afternoon hours, (**a**) Day 1, (**b**) Day 2. This data was taken on 26th April 2023, with a mean temperature of 14.3 °C.

In the evaluation of PV systems performance, a comparative analysis was conducted between the VBPV system and the VMPV system. The results, illustrated in Fig. 4a, b, present a compelling narrative on the efficacy of bifacial technology in solar energy capture throughout the day. Figure 4a delineates the power output patterns of both systems over a 24-h period. Notably, the VBPV system exhibited a pronounced increase in power generation during the early morning hours, from 5:30 to 9:00 AM, where a bifacial gain of 1.64 kWh was recorded. This trend was not an isolated incident; a similar surge was observed in the late afternoon window from 5:00 to 8:30 PM, with an additional gain of 1.39 kWh. Collectively, these increments contributed to a total daily power output of 24.57 kWh for the VBPV system, compared to 23.3 kWh for the VMPV system, marking a 1.27 kWh gain or a 7.87% improvement.

Complementing this, Fig. 4b reaffirms the superior performance of the VBPV system under what can be presumed to be varying operational conditions. The early morning hours once again showed an enhanced power output with a gain of 2.46 kWh, while the afternoon session contributed an additional 1.87 kWh. Collectively, these increments contributed to a total daily power output of 24.66 kWh for the VBPV system, compared to 22.85 kWh for the VMPV system, marking a 1.81 kWh gain or a 11.45% improvement.

The consistency with which the VBPV system outstripped the VMPV system in energy generation is a testament to the inherent advantages of bifacial technology. By effectively harnessing sunlight not only from direct overhead exposure but also from reflected light, the VBPV system demonstrates its capacity for increased energy capture, particularly during the low-angle sunlight periods at dawn and dusk. This ability to capitalize on diffuse and reflected irradiance adds a dimension of efficiency that is particularly advantageous in regions with significant ground albedo^21,24^ or in installations with reflective surroundings.

### Vertical bifacial PV vs tilted monofacial PV

**Figure 5:**
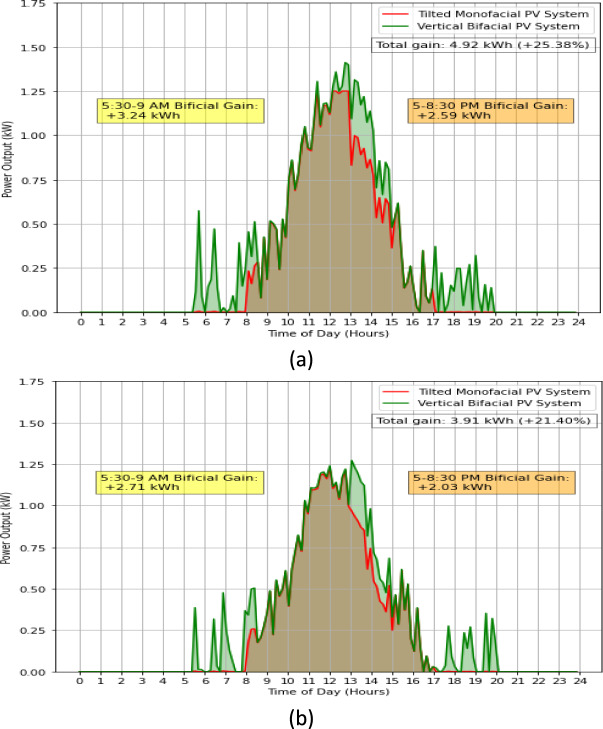
Comparative daily power output of VBPV versus TMPV Systems, (**a**) Day 1, (**b**) Day 2. This data was taken on 7^th^ May 2023, with a mean temperature of 16.7 °C.

Our comprehensive assessment extends to Fig. 5a, b, which provide further evidence of the enhanced performance of the VBPV system compared to the TMPV system. These figures represent a pivotal set of data showcasing the daily power output and clearly delineate the differential advantages offered by the bifacial technology under varied lighting conditions.

In Fig. 5a, we observe that the VBPV system significantly surpasses the TMPV system during the early hours, with a recorded bifacial gain of 3.24 kWh between 5:30 and 9:00 AM. This trend of increased efficiency extends to the latter part of the day, with an additional gain of 2.59 kWh noted from 5:00 to 8:30 PM. The aggregate gain for the VBPV system in this instance is an impressive 4.92 kWh, which equates to an enhancement of 25.38% when compared to its monofacial counterpart.

Similarly, Fig. 5b corroborates the superior performance of the bifacial system. The morning hours once again present a marked advantage with a bifacial gain of 2.71 kWh. The evening period contributes to this lead with a gain of 2.03 kWh. Together, these increases amount to a total gain of 3.91 kWh for the VBPV system, representing a 21.40% boost in power output over the TMPV system.

The substantial gains in power output during the less intense light conditions of morning and evening highlight the potential for VBPV systems to provide a more consistent energy supply throughout the day, mitigating the well-known midday peak in power generation associated with traditional solar systems. This distribution of energy generation could align more closely with typical consumption patterns, thereby enhancing the match between supply and demand. For instance, residential energy consumption typically peaks in the early morning and late afternoon to evening hours, coinciding with periods when people are at home and engaging in activities such as cooking, heating, and using electronic devices^37^. Similarly, commercial buildings experience peak energy demand in the late morning and early afternoon, driven by the operation of lighting, HVAC systems, and office equipment^38,39^. By aligning energy generation with these demand patterns, VBPV systems can improve grid stability and reduce the reliance on energy storage solutions or supplementary power sources.

### Monthly power gain comparison

**Figure 6:**
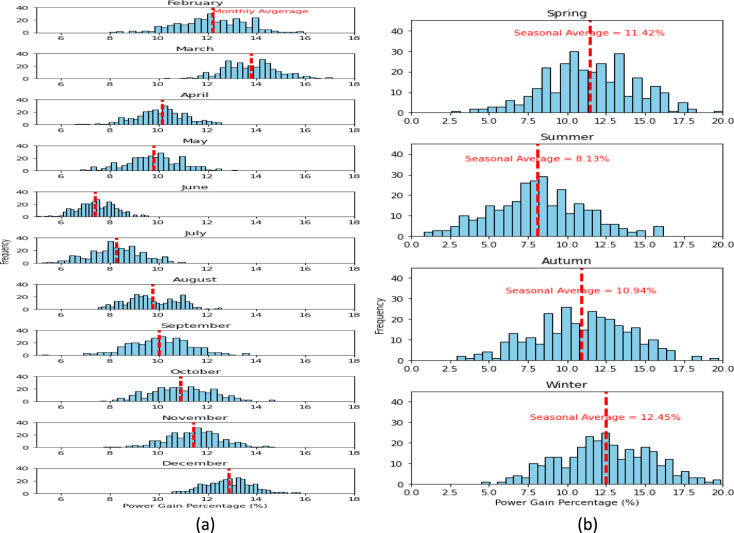
VBPV compared to VMPV. (**a**) Monthly power gain (Percentage, %) for VBPV over VMPV. (**b**) Seasonal variations in power gain (Percentage, %) for VBPV over VMPV. The histograms represent the frequency distribution of the power gain percentages, and the red dashed lines indicate the seasonal average power gains.

**Figure 7:**
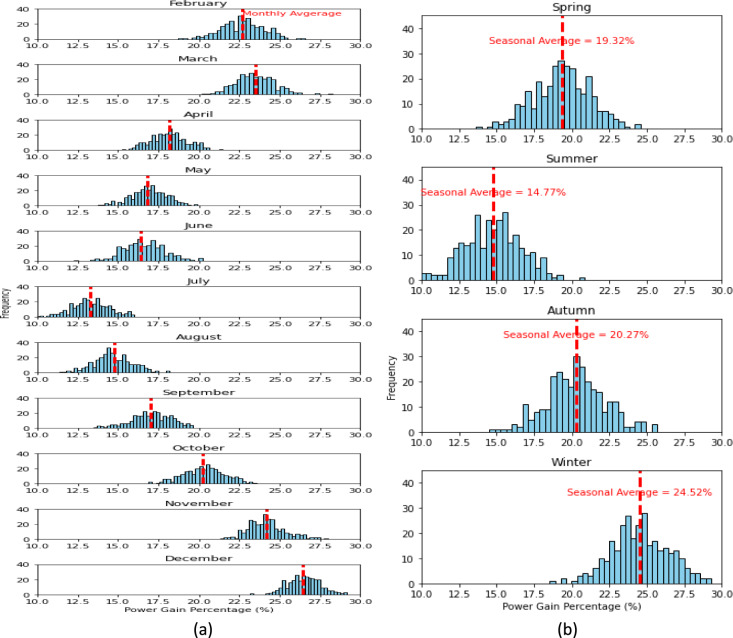
VBPV compared to TMPV. (**a**) Monthly power gain (Percentage, %) for VBPV over TMPV. (**b**) Seasonal variations in power gain (Percentage, %) for VBPV over TMPV. The histograms represent the frequency distribution of the power gain percentages, and the red dashed lines indicate the seasonal average power gains.

This section analyzes the performance enhancements of the VBPV system in comparison to both VMPV and TMPV systems, as depicted in Figs. 6 and 7, respectively. Figure 6 offers a nuanced view of the monthly power gains achieved by the VBPV system over the VMPV system, categorized by season. The histograms detail the frequency of power gain percentages, with a red dashed line indicating the seasonal average. In spring, the VBPV system shows a robust average power gain of 11.42%, indicating its superior performance during a time when sun angles and daylight hours start to increase. Summer, typically characterized by high solar irradiance, presents an average gain of 8.13%, a figure that might reflect high baseline performance from the VMPV system, reducing the relative gain. Autumn and winter follow with average gains of 10.94% and 12.45%, respectively, illustrating the VBPV system's effective light capture even during seasons with lower solar angles and shorter daylight hours.

Turning to Fig. 7, the VBPV system's performance is compared with the TMPV system. Here, the seasonal average power gains are significantly higher, underscoring the VBPV system's advanced capabilities. Spring shows a remarkable average gain of 19.32%, indicating the profound impact of bifacial technology during this season. Summer months present an average gain of 14.77%, autumn shows a substantial 20.27%, and winter peaks with a 24.52% average gain, reinforcing the idea that the VBPV system's design is particularly beneficial in capturing low-angle light and diffused reflections, a common scenario in the colder months.

The data from Figs. 6 and 7 underscore the VBPV system's consistent and significant outperformance relative to both the VMPV and TMPV systems across all seasons. The marked efficiency of the VBPV system is reflective of its dual-capture capability, which enables it to harness light from both its front and rear surfaces. This capability is evidenced in the results by the substantial power gains observed during periods of diffuse light conditions, such as early morning and late afternoon, as well as during seasons with lower sun angles, like autumn and winter. Specifically, the VBPV system's ability to capture reflected light from the ground and surrounding surfaces significantly contributes to its enhanced performance, as demonstrated by the higher average power gains in comparison to monofacial systems. This dual-capture feature ensures that the VBPV system maximizes energy harvest from both direct sunlight and diffuse, reflected light, leading to a more consistent and higher overall energy output.

**Figure 8:**
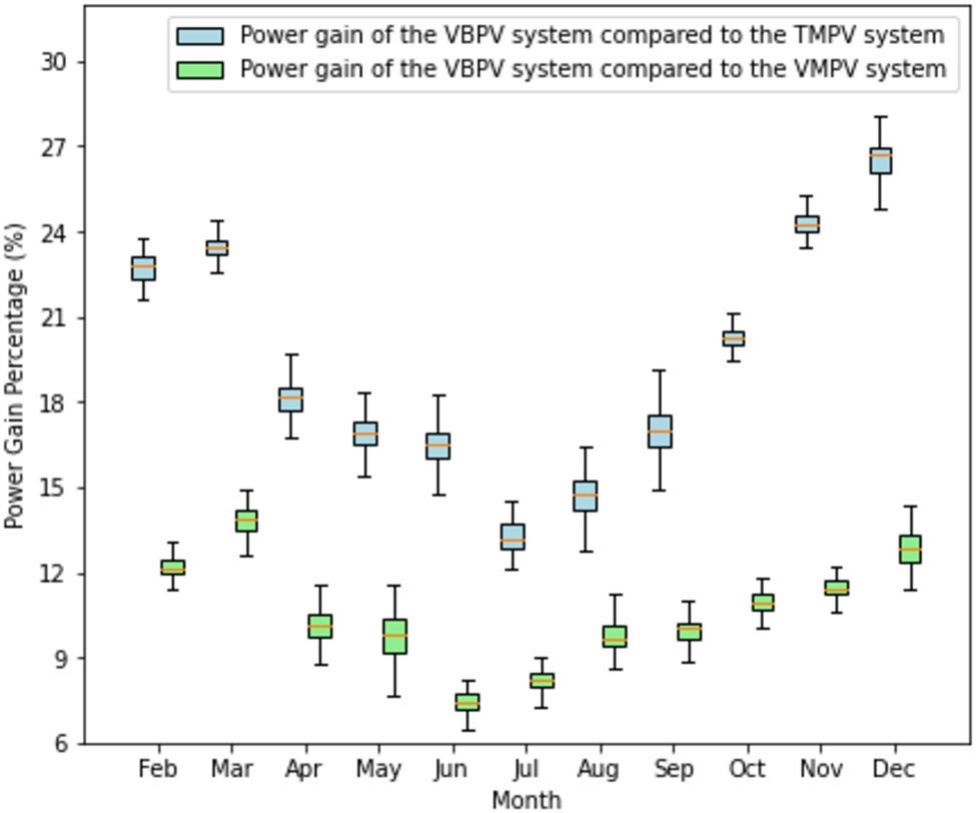
Annual comparative analysis of monthly power gain percentages for VBPV versus TMPV and VBPV versus VMPV systems. The box plots illustrate the distribution of monthly power gain percentages for each system throughout the year. The blue box plot shows the power gain of the VBPV system compared to the TMPV system, while the green box plot shows the power gain of the VBPV system compared to the VMPV system. Median values are indicated by the horizontal lines within each box.

In concluding to this section, Fig. 8 offers a comprehensive statistical overview of the PV systems over an annual cycle. The box plot visualization encapsulates the monthly power gain percentages, delivering a succinct and robust comparative analysis. The box plots reveal that the VBPV system consistently exhibits higher power gains when compared to the TMPV and VMPV systems throughout the year. These gains are quantified by the median of each box, indicating that regardless of the month, the VBPV system capitalizes on its design, which allows it to capture additional energy from reflected light not accessible to monofacial systems.

A critical observation from Fig. 8 is that the VBPV system not only outperforms the TMPV but also shows a significant advantage over the VMPV system. This distinction is noteworthy as it suggests that the enhancements in bifacial technology translate to tangible gains in power output, even when compared to a more conventional monofacial system like the VMPV. When analyzing the VBPV's performance against the TMPV system, we see an even more pronounced difference in reflective gain. The box plots for the VBPV and TMPV comparison stretch higher on the percentage axis, indicating that the traditional system, without the advanced technology of the VMPV, falls short in harnessing the available solar energy. Moreover, the box plots for the VBPV and VMPV comparison demonstrate that the VMPV, while more efficient than the TMPV, cannot match the VBPV system's capacity for increased energy capture. This pattern is consistent across all months, underlining the VBPV's superior design and efficiency.

**Figure 9:**
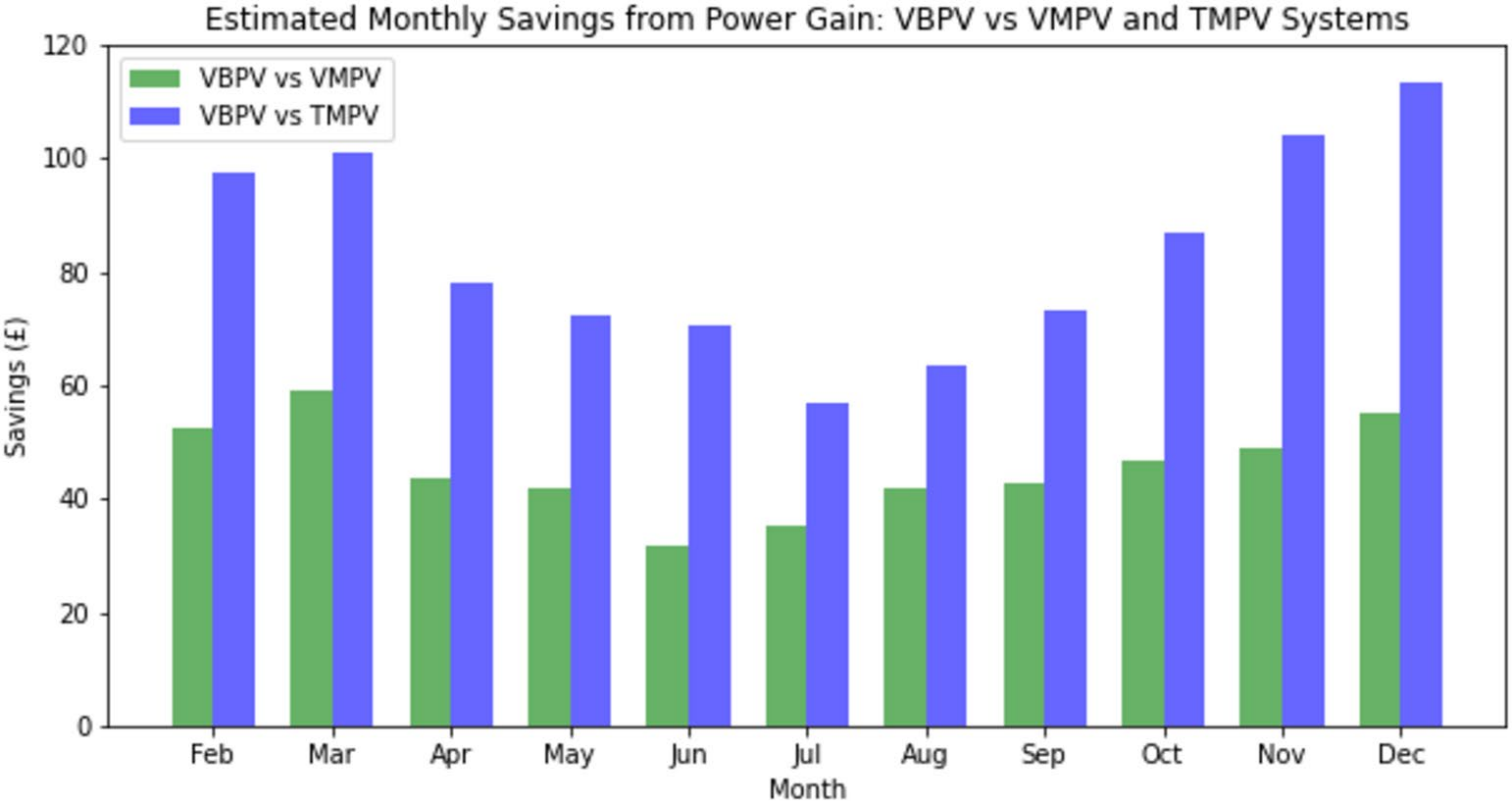
Comparative Estimation of Monthly Savings Achieved Through Power Gain: A side-by-side comparison of the economic advantages of using VBPV systems versus VMPV systems (in green) and TMPV systems (in blue), across each month of the year.

To ascertain the financial benefits of VBPV systems, we conducted an analysis based on the monthly power gain percentages derived from empirical data, taken from Fig. 8. Using an assumed standard monthly energy output of 1500 kWh as a baseline for all the systems, we applied the power gain percentages to estimate the additional energy produced solely due to the bifacial gain. The cost of electricity was factored in at the 2023 standard variable price of 28.62p/kWh. This price point reflects the retail electricity rate for an average consumer in the UK, which is subject to regional variations and market fluctuations. The analysis revealed discernible monthly fluctuations in savings (as shown in Fig. 9), which correspond with the changes in power gain percentages over the course of the year. The savings reached their zenith during the summer months, in alignment with the augmented power gains from increased solar irradiance. Conversely, the savings diminished during the winter months, reflecting the diminished solar irradiance inherent to the season.

For the VBPV compared with the VMPV systems, the additional solar energy captured by the bifacial technology translated into considerable monthly and cumulative annual savings. With the power output for these systems set at 1500 kWh, the use of VBPV systems resulted in a total estimated annual saving of £932.58 over the VMPV systems (Fig. 9). These savings are reflective of the consistent additional power generation offered by VBPV systems across all months, with the highest gains observed during the peak solar irradiance months of summer. In comparison to the TMPV systems, the VBPV systems demonstrated even greater economic advantages. The enhanced power gain percentages of VBPV systems, particularly noted during the winter months, emphasize their efficiency in low-irradiance conditions. The annual savings when comparing VBPV to TMPV systems amounted to a notable £1,221.13. This significant difference in savings highlights the VBPV system's ability to harness solar energy more effectively throughout the year, including during periods of lower sunlight availability.

In addition to the power gain analysis, a cost estimation comparison between the VBPV, VMPV, and TMPV systems is provided. The analysis considers the initial installation costs, maintenance costs, and the economic benefits derived from the increased energy output of the VBPV system. The initial installation cost of the VBPV system is higher than that of the VMPV and TMPV systems due to the advanced bifacial technology and the need for specialized mounting structures. Based on current market prices, the estimated cost per kW for VBPV systems is approximately £1,200, compared to £1,000 for VMPV and £900 for TMPV systems. Maintenance costs for VBPV systems are slightly lower due to the reduced accumulation of dirt and debris on vertically mounted panels.

To provide a comprehensive economic comparison, the annual energy savings and return on investment (ROI) were calculated. The cost of electricity in the UK is approximately £0.2862 per kWh. The annual additional energy produced by the VBPV system, as demonstrated in Fig. 9, results in significant cost savings compared to VMPV and TMPV systems.

### Bificail PV system gain vs solar irradiance

This section presents a critical analysis of the modeling challenges and successes encountered in simulating the performance of bifacial PV systems. Plane of Array (POA) irradiance, which refers to the solar irradiance incident on the plane of the PV array, is a key parameter in this analysis. However, to provide a complete picture of the relations, both direct and diffuse irradiance contributions to the bifacial gain are compared.

**Figure 10:**
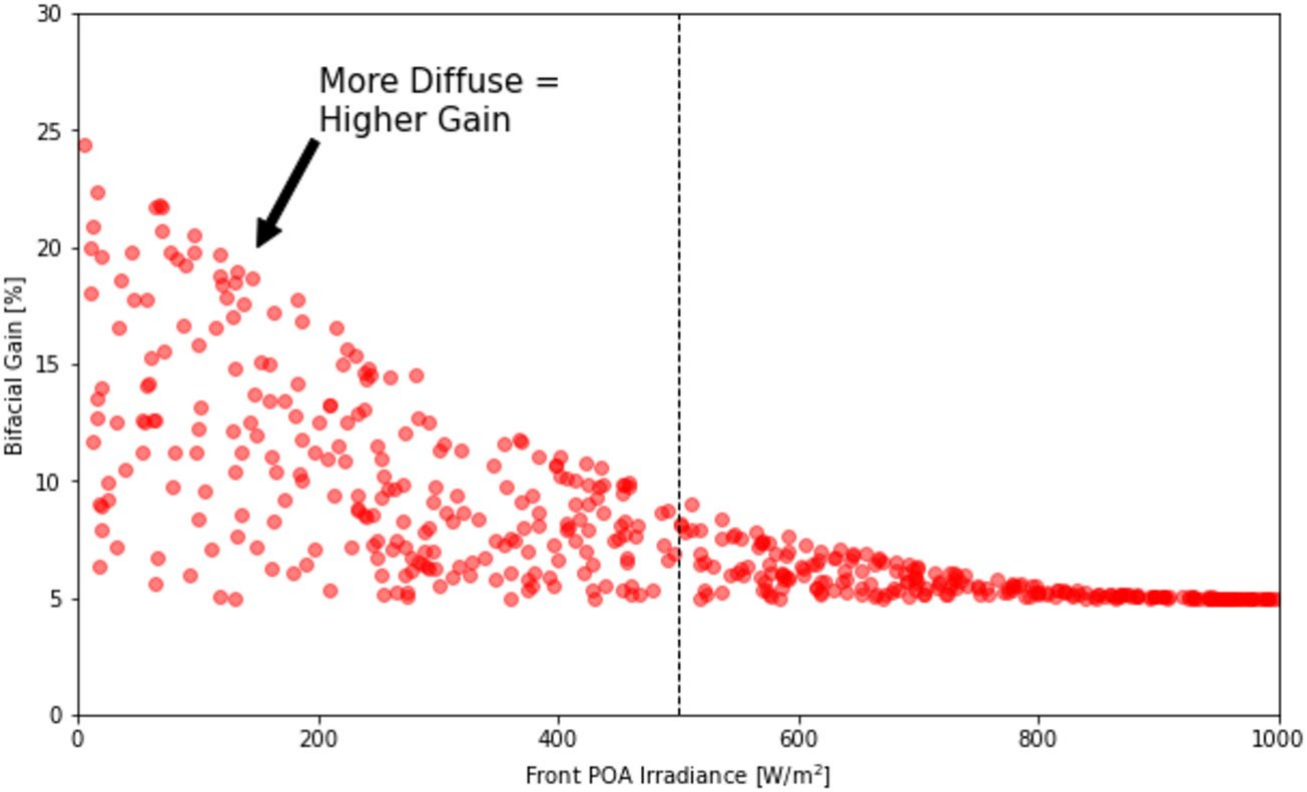
Correlation between bifacial gain and diffuse irradiance, highlighting the importance of diffuse light in bifacial PV system performance. The scatter plots show data points and regression lines indicating the trend, highlighting the significant role of diffuse irradiance in bifacial PV system performance.

Figure 10 illuminates the relationship between bifacial gain and incident light, showcasing a clear trend where increased diffuse irradiance correlates with higher bifacial gain. This direct association highlights the complex interplay between light conditions and the energy capture efficiency of bifacial panels^7^. The scatter of data points emphasizes the difficulty in predicting performance due to the variability of solar irradiance, especially the proportion of diffuse light^40^. Such insights indicate that current modeling approaches may need refinement to account for this variability. This complexity is further evidenced by the limited data available for bifacial systems, which constrains the ability of models to accurately capture the nuances of their performance. The scarcity of robust datasets is a significant hurdle, suggesting a pressing need for more comprehensive data collection to improve the predictability and reliability of bifacial PV system models.

**Figure 11:**
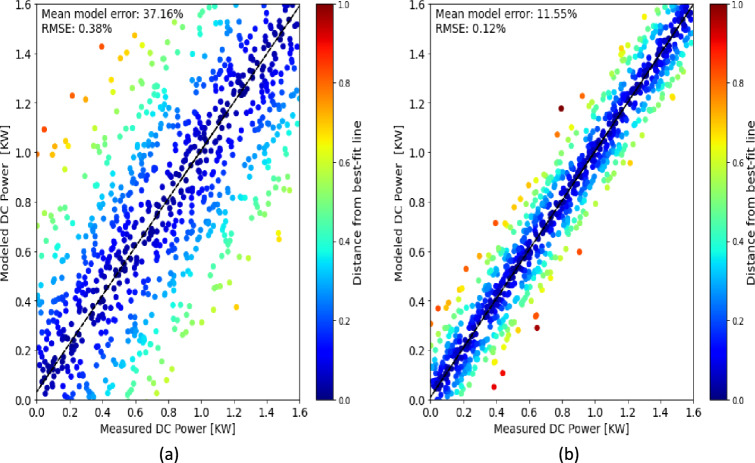
Modelling VBPV system output power (mix between hourly and daily data samples), (**a**) Initial modelling results, (**b**) Refined modelling results with adjusted sunlight variability.

Transitioning to Fig. 11a, we examine the initial modeling attempts using the SAM NREL model^41,42^, which did not adequately capture the performance of the VBPV system. The figure portrays a significant discrepancy between modeled DC power and measured DC power, evidenced by the mean model error of 37.16% and an RMSE of 0.38%. This gap between expected and actual performance underscores the limitations of the model when it does not incorporate critical factors such as the variability of sunlight, particularly the diffuse component.

In the quest to enhance the fidelity of PV system performance models, the incorporation of sunlight variability, specifically the ratio of diffuse to direct sunlight, stands as a pivotal aspect. This is particularly crucial for bifacial PV systems due to their ability to capture light from both their front and rear sides. The ratio of diffuse to direct sunlight can dramatically influence the amount of light received by the rear side of bifacial panels, which is not directly exposed to the sun. For this reason, Fig. 11b presents a refined modeling approach where the variability of the sun, especially the ratio of diffuse to direct sunlight, is accounted for. The adjusted model results in a markedly improved correlation between modeled and measured DC power, with a substantially reduced mean model error of 11.55% and an RMSE of 0.12%. This improved alignment validates our hypothesis that incorporating the dynamic nature of sunlight, and its interactions with bifacial panels, is essential to accurately simulate their performance.

The refined model can be described by a set of equations that account for the bifacial gain, which is a function of both the direct and diffuse components of solar irradiance. The ratio of diffuse to direct irradiance, also known as the clearness index, is a crucial parameter in evaluating the performance of bifacial PV systems. This ratio, widely reported in the literature, indicates the proportion of solar radiation that is diffuse as opposed to direct. A higher clearness index signifies more diffuse light, which is particularly advantageous for bifacial systems as they can capture light from both their front and rear surfaces. According to^43^, understanding the clearness index is essential for accurately modeling bifacial PV performance, as it affects the amount of light available for the rear side of the panels. Similarly^44^, emphasized that regions with higher diffuse irradiance ratios exhibit enhanced bifacial gains. These findings underscore the importance of incorporating the clearness index in performance models for bifacial PV systems.

Let ${G}_{bifacial}$ be the bifacial gain, ${I}_{direct}$ is the direct irradiance, ${I}_{diffuse}$ is the diffuse irradiance, therefore, the bificail gain can be calculated in (2).

2 $${G}_{bifacial}=\frac{{I}_{direct}\left(1+\propto {R}_{ground}\right)+{I}_{diffuse}(1+\beta {R}_{sky})}{{I}_{direct}+{I}_{diffuse}}$$

where $\propto$ is the bifaciality coefficient for ground-reflected irradiance, ${R}_{ground}$ is the ground albedo, $\beta$ is the bifaciality coefficient for sky-diffuse irradiance, and ${R}_{sky}$ is a factor representing the effective sky view factor affecting diffuse irradiance capture. The total amount of power output, ${P}_{modelled}$, can then be calculated by (3). Where ${P}_{STC}$ is the power output under standard test conditions, ${\eta }_{conversion}$ is the conversion efficiency of the PV cells, and $FF$ is the fill factor.

3 $${P}_{modelled}={G}_{bifacial} \times {P}_{STC}\times {\eta }_{conversion}\times FF$$

To calibrate the model with respect to the ratio of diffuse to direct sunlight, we introduce weighting coefficients that adjust the impact of each component on the total irradiance. The calibration process involves optimizing these coefficients so that the model output matches measured data as closely as possible. This was achieved by adjusting, ${w}_{direct}$ and ${w}_{diffuse}$, the weighting coefficients for direct and diffuse irradiance, respectively. And therefore, to find the total effective irradiance, ${I}_{effective}$ calculated using (4). The optimization process aims to find the values of ${w}_{direct}$ and ${w}_{diffuse}$, that minimize the error between the modeled and measured power output. This was achieved using an Levenberg–Marquardt optimization algorithm^45^, which is suited for solving non-linear least squares problems^46^.

4 $${I}_{effective}=({w}_{direct}{ \times I}_{direct})+{(w}_{diffuse}{\times I}_{diffuse})$$

**Figure 12:**
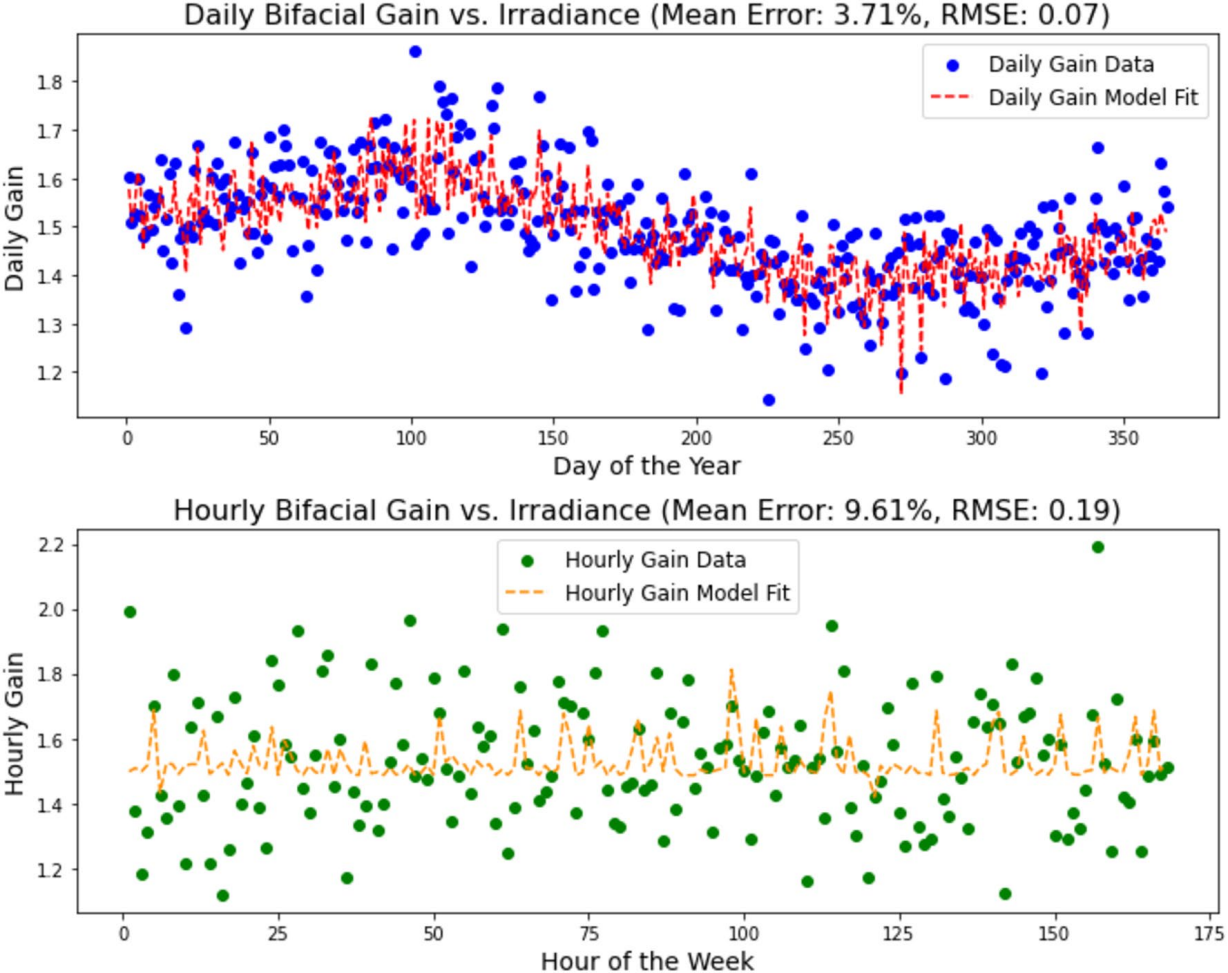
Comparative analysis of bifacial gain vs. irradiance on daily and hourly basis. The top panel illustrates the variation and model fit of daily bifacial gain over a year, while the bottom panel depicts the hourly bifacial gain for a week. The polynomial model fits (red dashed line for daily data, orange dashed line for hourly data) highlight the challenge in capturing temporal dynamics in bifacial PV system performance.

Figure 12 presents the outcomes of modelling bifacial gain versus irradiance over two distinct temporal scales: daily and hourly. In the top panel, showcasing daily data, we observe the daily bifacial gain plotted against the day of the year. The data points, marked in blue, display a degree of variability that seems to follow a seasonal trend, likely reflecting the sinusoidal nature of solar irradiance throughout the year. A polynomial model fit, depicted by the red dashed line, attempts to capture this underlying trend. The fit seems to trace the central tendency of the data but does not adhere closely to individual data points, reflecting in a mean model error of 3.71% and an RMSE of 0.07. These metrics suggest that while the model grasps the general pattern, there is room for improvement, particularly in capturing the daily variability.

The bottom panel of Fig. 12 displays the hourly data, where each green dot represents the hourly bifacial gain for a particular hour of the week. Here, the volatility is more pronounced, reflecting the more dynamic changes in irradiance that occur throughout the day. The hourly model fit, illustrated by the orange dashed line, shows considerable deviation from the actual data points, with a mean error of 9.61% and an RMSE of 0.19. This discrepancy indicates that the hourly variations in irradiance and corresponding bifacial gain are not adequately captured by the current model, suggesting a need for a more complex or different modeling approach for short-term predictions.

## Discussion

The environmental and economic implications of adopting VBPV systems on a large scale are multifaceted and far-reaching. Environmentally, the most significant impact would be the substantial reduction in carbon emissions. Solar power is a clean, renewable resource, and the increased efficiency of VBPV systems means that more electricity can be generated per unit area compared to traditional solar solutions. This increased efficiency is critical in densely populated or land-scarce regions where the optimization of limited space is essential. Furthermore, the dual-sided nature of bifacial panels captures reflected light, enhancing energy yield and reducing the need for additional land, which is crucial for preserving natural habitats and biodiversity. These findings are consistent with studies that highlight the environmental benefits of bifacial PV systems, such as reduced land use^47^ and lower carbon footprint^48^.

From an economic standpoint, the adoption of VBPV systems could lead to substantial cost savings over time. Although the initial investment might be higher than traditional systems due to the advanced technology involved, the higher energy yield and efficiency of VBPV systems will likely result in lower long-term costs. According to recent studies, bifacial PV systems can provide a return on investment that is 20–30% higher compared to monofacial systems due to the additional energy captured from the rear side^47,48^. Additionally, the maintenance costs might be lower due to the vertical design, which is less prone to dirt accumulation and potential shading issues. This factor alone could make VBPV systems more economically viable, especially in regions where labour and maintenance costs are significant factors.

The findings of this study have profound implications for global renewable energy strategies. The enhanced efficiency of VBPV systems aligns well with the growing global emphasis on sustainable development and the urgent need to shift to renewable energy sources. Studies have demonstrated the viability of bifacial PV systems in various urban environments, highlighting their adaptability and high energy yield even in constrained spaces^47^. For instance, bifacial PV installations on building facades and rooftops have shown significant energy production benefits^49^, supporting the transition to more sustainable urban infrastructure. By demonstrating the potential of VBPV systems in diverse environmental settings, this technology could play a pivotal role in the transition to a low-carbon economy.

In terms of policy and planning, these findings could influence government and industry leaders to reconsider their investment strategies. Encouraging the adoption of VBPV technology in urban planning and building design could be a significant step towards achieving energy efficiency targets. The literature since 2018 has explored various aspects of bifacial PV systems, emphasizing their efficiency, cost-effectiveness, and integration into smart grids such^50,51^. Future research should focus on testing VBPV systems in a variety of geographical locations and environmental conditions to validate and extend these findings. Additionally, it would be beneficial to explore the integration of VBPV systems with other renewable energy technologies such as wind or hydroelectric power to create more robust and resilient energy systems.

The specific geographical location and environmental conditions of York, UK, where this study was conducted, play a significant role in the performance of VBPV systems. York experiences a temperate maritime climate, characterized by relatively mild temperatures throughout the year, moderate rainfall, and variable cloud cover. The average annual temperature is around 10°C, with average daylight hours ranging from approximately 5–7 h in winter to 14–16 h in summer. The sun angle in York varies significantly with the seasons, reaching a maximum elevation of about 62 degrees during the summer solstice and a minimum of approximately 15 degrees during the winter solstice. These climatic conditions and solar geometry are critical factors influencing the performance of VBPV systems, as they determine the amount of direct and diffuse irradiance received by the panels.

In summary, the environmental and economic potential of VBPV systems is significant, with the possibility to make a considerable impact on global renewable energy strategies. However, acknowledging and addressing the limitations of current research is crucial in advancing this technology and maximizing its benefits.

## Conclusions

This pioneering study on the VBPV system marks a significant leap forward in the realm of solar energy technology. Our comprehensive year-long research at the University of York, UK, serves as the first in-depth exploration of this innovative concept, diverging from conventional solar panel installations. The VBPV system, with its vertical orientation and utilization of advanced HJT cells, has demonstrated exceptional performance, surpassing traditional solar solutions in efficiency and energy output.

Key findings of this study reveal the superior capability of the VBPV system compared to its counterparts. Notably, the system outperformed VMPV system, showing a 7.12% and 10.12% increase in daily power output during early morning and late afternoon periods. When compared to a traditional TMPV system, the VBPV system exhibited even more remarkable gains, with a 26.91% and 22.88% enhancement in energy output in similar time frames. Seasonal analysis further highlights the system's efficiency, with average power gains of 11.42% in spring, 8.13% in summer, 10.94% in autumn, and 12.45% in winter over the VMPV system. Against the TMPV system, these gains peaked at an impressive 24.52% in the winter months.

These findings underscore the VBPV system's unparalleled ability to harness solar energy efficiently, irrespective of seasonal variances. Its design not only maximizes land use but also integrates seamlessly with modern architectural landscapes, adding an aesthetic value to its functional benefits. The system's bifacial technology, capable of capturing solar radiation from both sides, significantly boosts its energy yield, making it a potent solution for regions with variable sun exposure and reflective environments.

In conclusion, the VBPV system emerges as a promising solution for the future of sustainable energy. Its innovative design, superior efficiency, and adaptability to various environmental conditions position it as an ideal candidate for widespread adoption in both urban and rural settings. This study paves the way for future research and development in photovoltaic technology, encouraging a shift towards more efficient, environmentally friendly, and architecturally integrated solar energy solutions. As the first paper to delve into this new PV technology and concept design, it lays a strong foundation for the evolution of solar energy systems, steering the industry towards a more sustainable and energy-efficient future.

## Data Availability

Data will be made available on reasonable request to the corresponding author of the paper.

## Abbreviations

CFD: Computational fluid dynamics
DC: Direct current
HJT: Heterojunction
IBC: Interdigitated Back Contact
NREL: National Renewable Energy Laboratory
PERC: Passivated Emitter and Rear Cell
PERL: Passivated Emitter Rear Locally Diffused
PERT: Passivated Emitter Rear Totally Diffused
POA: Plane of Array
PV: Photovoltaic
RMSE: Root mean square error
STC: Standard test conditions
TMPV: Tilted monofacial photovoltaic
VBPV: Vertical bifacial photovoltaic
VMPV: Vertical monofacial photovoltaic
